# Supplementary material for: Gene duplication and deletion caused by over-replication at a fork barrier
Source: Nat Commun. 2023 Nov 25;14:7730. doi: 10.1038/s41467-023-43494-7 (PMC10676400; doi:10.1038/s41467-023-43494-7)
Supplement: Supplementary file 1 — Supplementary Information [file 41467_2023_43494_MOESM1_ESM.pdf]

Supplementary information

Gene duplication and deletion caused by over-replication at a fork barrier

Judith Oehler[1,2], Carl A. Morrow[1,2] and Matthew C. Whitby[1]*

[1]Department of Biochemistry, University of Oxford,

South Parks Road, Oxford, OX1 3QU, UK

[2]These authors contributed equally

*Correspondence: matthew.whitby@bioch.ox.ac.uk

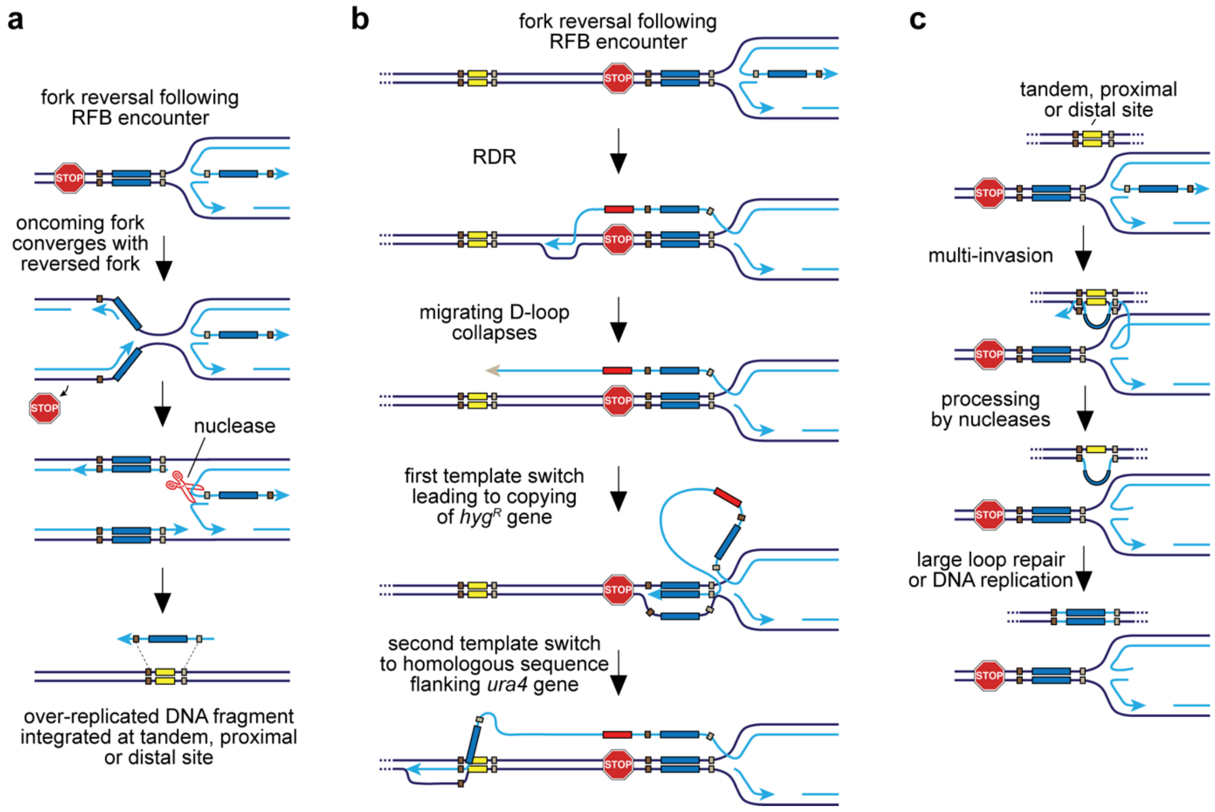

Supplementary Figure 1. Potential models for Dup-Del formation

a DRFT. **b** RDR-associated template switching. **c** Rad51-mediated multi-invasion from a reversed replication fork. In each model, the over-replicated DNA encompasses a gene (blue box) flanked by interspersed repetitive DNA sequences (brown and grey boxes). Parental DNA strands are in dark blue and nascent strands are in light blue. Relevant 3' DNA ends are indicated by the light blue arrowheads. The RFB is indicated by the red stop symbol.

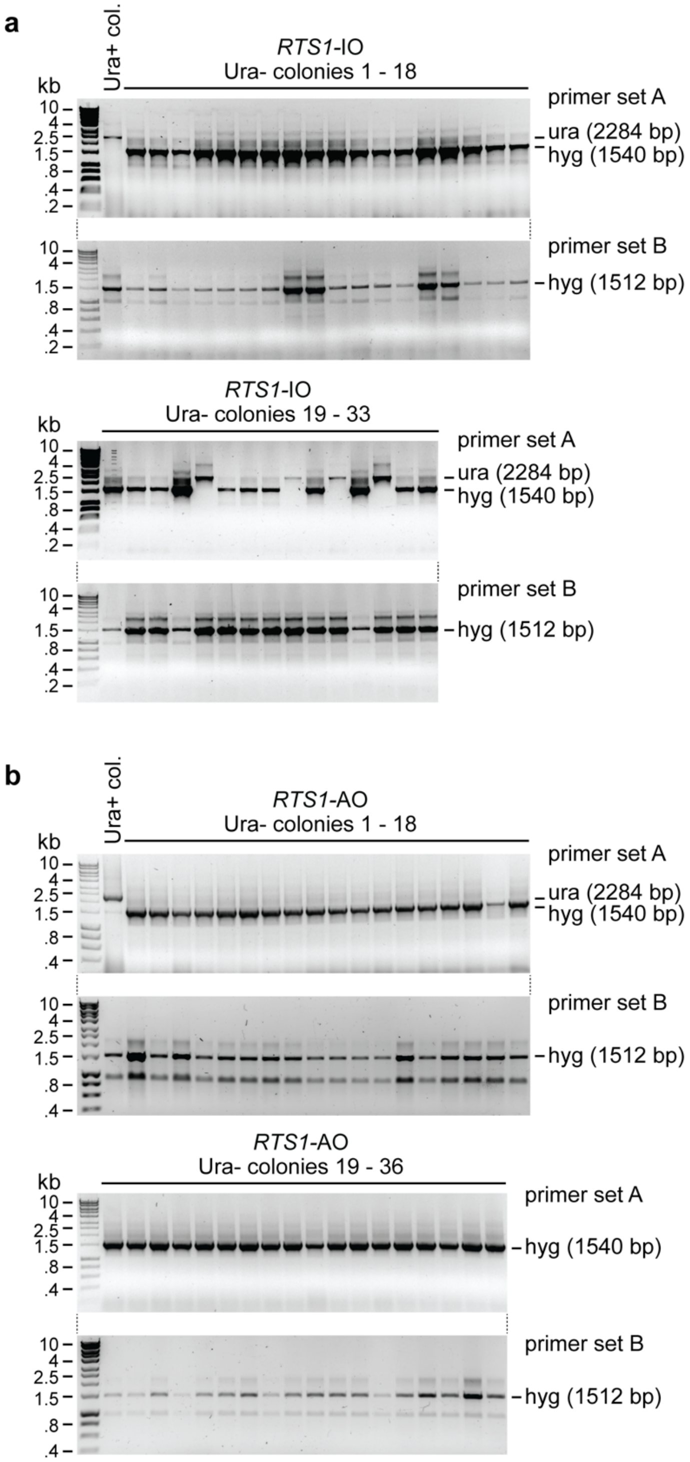

Supplementary Figure 2. PCR analysis of Ura- (FOA resistant) colonies

a PCR analysis of genomic DNA purified from 33 independent Ura- colonies and one Ura+ colony from a wild-type strain (MCW9374) containing *RTS1*-IO and the Dup-Del reporter shown in Figure 1a. The location of primer sets A and B are shown in Figure 1a. **b** PCR analysis of genomic DNA purified from 36 independent Ura- colonies and one Ura+ colony from a wild-type strain (MCW9235) containing *RTS1*-AO and the Dup-Del reporter shown in Figure 1a. The location of primer sets A and B are shown in Figure 1a. Source data are provided as a Source Data file.

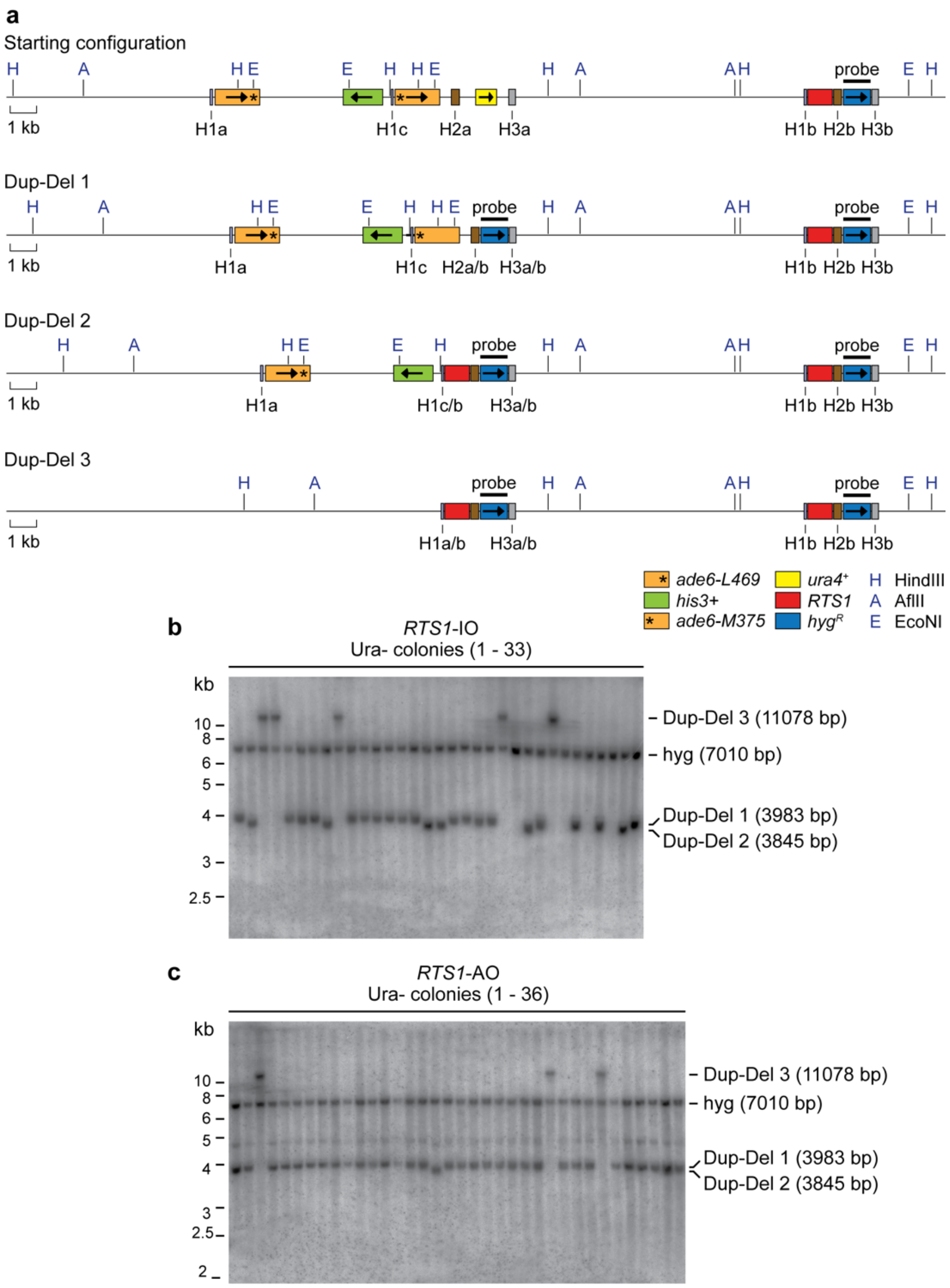

Supplementary Figure 3. Southern blot analysis of Ura- (FOA resistant) colonies

a Restriction map of genomic region encompassing the Dup-Del reporter. The starting configuration of the reporter and three classes of Dup-Del rearrangement are shown. The H1b sequence shares 113 bp of homology with H1a and 92 bp with H1c. In both cases the homology is interrupted by two base-base mismatches within 20 bp of their telomere proximal end. H2a and H2b share a 393 bp stretch of perfect homology, and H3a and H3b share 249 bp of homology interrupted by one base pair mismatch and a 3-nucleotide insertion/deletion loop at their telomere proximal end (see Supplementary Fig. 5). **b** Southern blot analysis of the same 33 Ura- colonies analysed by PCR in Supplementary Figure 2a. The genomic DNA was digested with HindIII, and restriction fragments were detected using *hyg*^{R} as the probe. **c** Southern blot analysis of the same 36 Ura- colonies analysed by PCR in Supplementary Figure 2b. The genomic DNA was digested with HindIII, and restriction fragments were detected using *hyg*^{R} as the probe. Source data are provided as a Source Data file.

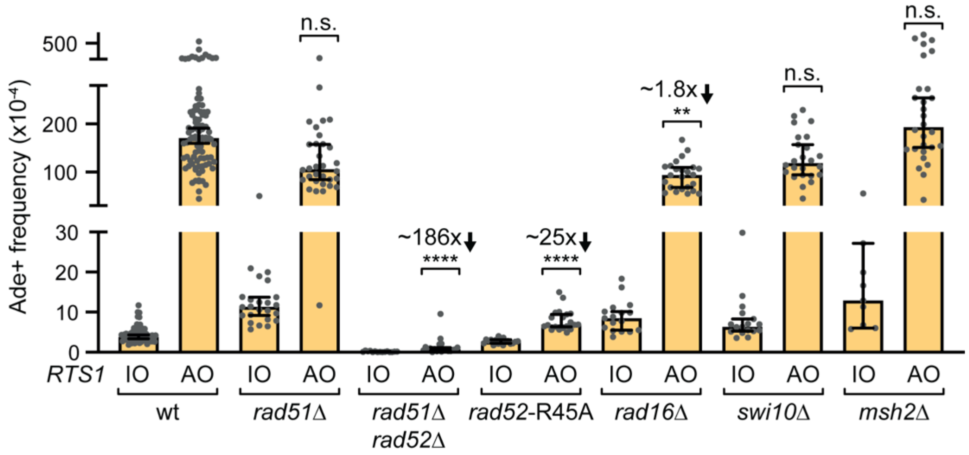

Supplementary Figure 4. RDR-associated template switching does not depend on Rad16-Swi10 or Msh2

Frequency of spontaneous (*RTS1*-IO) and *RTS1*-AO-induced Ade+ recombinants in the indicated strains. The recombination reporter is shown in Figure 2a. Fold changes are relative to the *RTS1*-AO wild-type (wt) strain. Data are presented as median values ± 95% confidence interval with individual data points shown as grey dots. P-values are for the comparison to the *RTS1*-AO wild-type strain and were calculated by the Kruskal-Wallis test with Dunn's multiple comparisons post-test. **** p-value <0.0001; ** p-value 0.0054; n.s. not significant (*rad51*Δ p-value = 0.2398; *swi10*Δ p-value >0.9999; *msh2*Δ p-value >0.9999). The data are also reported in Supplementary Data 1, which includes the strain numbers, the number of colonies tested for each strain (*n*) and p-values. Further details of the statistical analysis are reported in Supplementary Data 2. Source data are provided as a Source Data file.

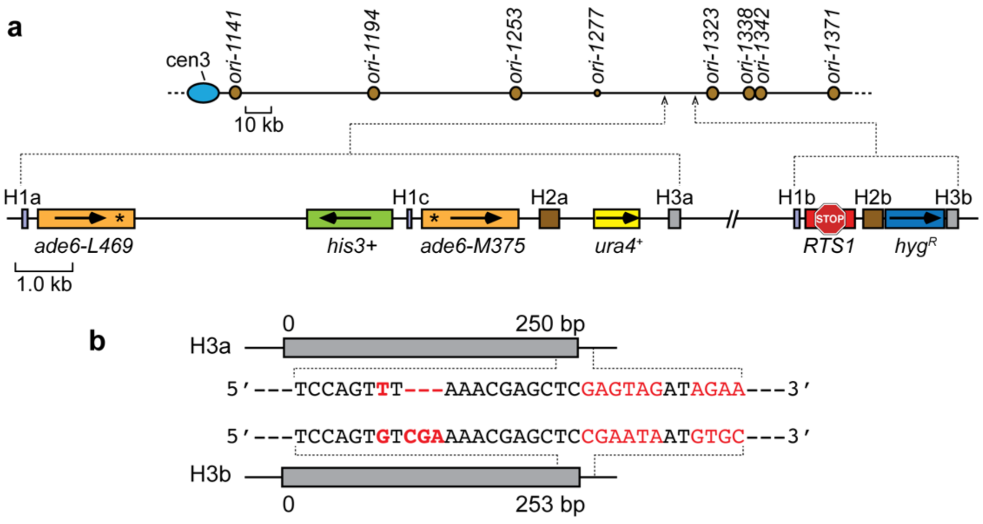

Supplementary Figure 5. Base-Base mismatch and IDL at the 3' end of H3a and H3b

a Diagram showing the Dup-Del reporter and its location on chromosome 3. Replication origins are indicated by the brown circles. The marker genes are indicated by the orange, green, yellow and blue rectangles with the arrows indicating the direction of transcription. The asterisks in *ade6-L469* and *ade6-M375* indicate the position of loss-of-function mutations. The *RTS1* RFB is indicated by the red stop symbol. The H1a/b/c, H2a/b and H3a/b sequences are indicated by the purple, brown and grey boxes, respectively. **b** Comparison of the H3a and H3b sequences showing the single base-base mismatch and 3 nucleotide IDL at their 3' end. Identical bases are in black and those that differ are in red.

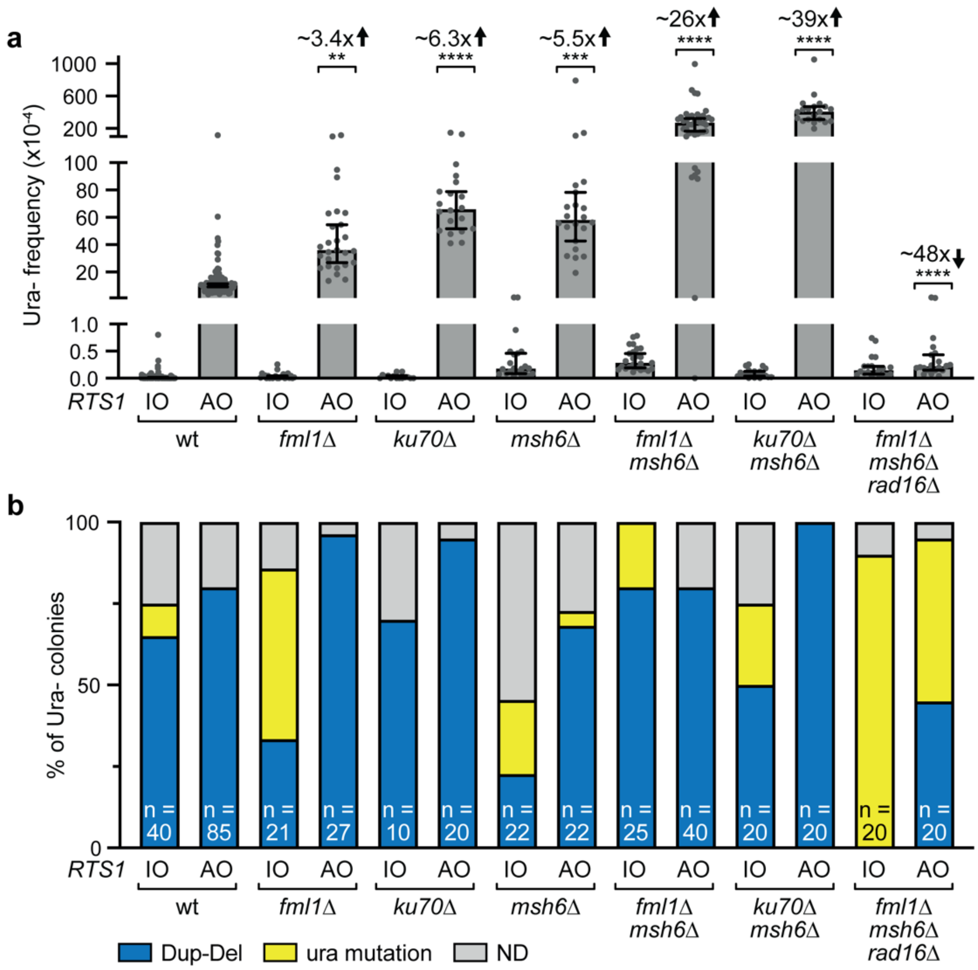

Supplementary Figure 6. Ku70 and Fml1 suppress Dup-Del formation both in the presence and absence of Msh6

a Frequency of spontaneous (*RTS1*-IO) and *RTS1*-AO-induced Ura- (FOA resistant) colonies in the indicated strains. Fold changes are relative to the *RTS1*-AO wild-type strain. Data are presented as median values ± 95% confidence interval with individual data points shown as grey dots. P-values are for the comparison to the *RTS1*-AO wild-type strain and were calculated by the Kruskal-Wallis test with Dunn's multiple comparisons post-test. **** p-value <0.0001; *** p-value 0.0003; ** p-value 0.003. The data are also reported in Supplementary Data 1, which includes the strain numbers, the number of colonies tested for each strain (*n*) and p-values. Further details of the statistical analysis are reported in Supplementary Data 2. **b** Percentage of FOA resistant colonies containing a Dup-Del or putative mutation in *ura4*/*ura5* determined by PCR. The data in Panel **b** relates to the data in Panel **a**. ND indicates a failure of one or both diagnostic PCRs. n indicates the number of independent FOA resistant colonies tested. Source data are provided as a Source Data file.

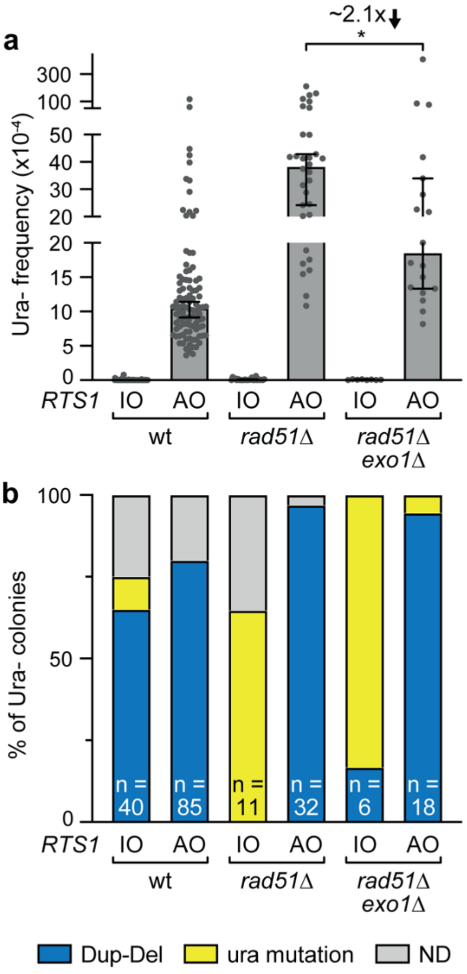

Supplementary Figure 7. Heightened levels of Dup-Dels in a *rad51*Δ mutant are partly dependent on Exo1

a Frequency of spontaneous (*RTS1*-IO) and *RTS1*-AO-induced Ura- (FOA resistant) colonies in the indicated strains. Data are presented as median values ± 95% confidence interval with individual data points shown as grey dots. The p-value for the comparison between wild-type and *exo1*Δ was calculated by the Kruskal-Wallis test with Dunn's multiple comparisons post-test. The p-value for the comparison between *rad51*Δ and *rad51*Δ *exo1*Δ was calculated by the Mann-Whitney test (two-tailed). * p-value 0.0207; n.s. not significant (p-value >0.9999). The data are also reported in Supplementary Data 1, which includes the strain numbers, the number of colonies tested for each strain (*n*) and p-values. Further details of the statistical analysis are reported in Supplementary Data 2. **b** Percentage of FOA resistant colonies containing a Dup-Del or putative mutation in *ura4*/*ura5* determined by PCR. The data in Panel **b** relates to the data in Panel **a**. ND indicates a failure of one or both diagnostic PCRs. n indicates the number of independent FOA resistant colonies tested. Source data are provided as a Source Data file.

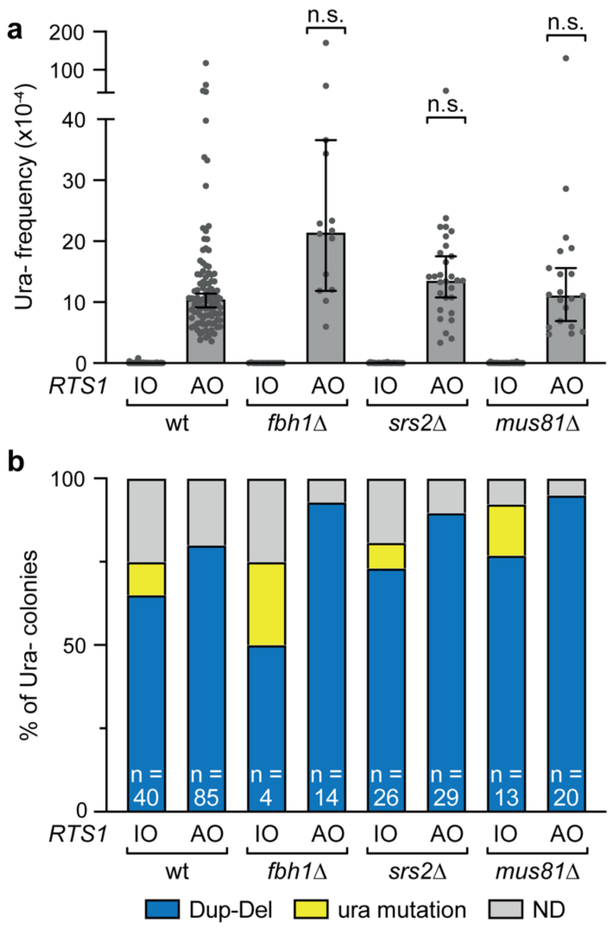

Supplementary Figure 8. *RTS1*-AO-induced Dup-Del formation is neither promoted nor constrained by Fbh1, Srs2 or Mus81

a Frequency of spontaneous (*RTS1*-IO) and *RTS1*-AO-induced Ura- (FOA resistant) colonies in the indicated strains. Data are presented as median values ± 95% confidence interval with individual data points shown as grey dots. P-values were calculated by the Kruskal-Wallis test with Dunn's multiple comparisons post-test. n.s. not significant (p-value >0.9999). The data are also reported in Supplementary Data 1, which includes the strain numbers, the number of colonies tested for each strain (*n*) and p-values. Further details of the statistical analysis are reported in Supplementary Data 2. **b** Percentage of FOA resistant colonies containing a Dup-Del or putative mutation in *ura4*/*ura5* determined by PCR. The data in Panel **b** relates to the data in Panel **a**. ND indicates a failure of one or both diagnostic PCRs. n indicates the number of independent FOA resistant colonies tested. Source data are provided as a Source Data file.

Supplementary Table 1. Recombination frequencies

Relevant Genotype and Strain no.	*RTS1* orientation	Position of recomb-ination reporter[a]	Number of colonies analysed (*n*)	Frequency of Ade^+ recombinants amongst FOA resistant colonies (x 10^{-4}) [b]
wild-type MCW9235	AO	11.4 kb down-stream	19	156.25 (78.74 - 330.6)

[a] The recombination reporter is shown in Figure 1a.
[b] Median value with 95% confidence interval in parenthesis.

Supplementary Table 2. Recombination frequencies (related to Figure 7)

Relevant Genotype and Strain no.	*RTS1* orientation	Position of recomb-ination reporter[a]	Number of colonies analysed (*n*)	Ura^- Ade^+ recombinant frequency (x 10^{-4})		Ura^- Ade^- recombinant frequency (x 10^{-4})		Figure
				Median (95% CI)[b]	p-value[c]	Median (95% CI)[b]	p-value[c]	
wild-type MCW9631	IO	10.6 kb down-stream	29	0.002315 (0.000 - 0.006033)	-	0.000 (0.000 - 0.000)	-	7b, 7c, 7d
wild-type MCW9632	AO	10.6 kb down-stream	29	14.30 (7.841 - 17.71)	<0.0001[d]	0.5286 (0.2800 - 0.8889)	<0.0001[d]	7b, 7c, 7d

[a] The recombination reporter is shown in Figure 7a.
[b] The values in parentheses are the 95% confidence interval.
[c] Approximate p-values were calculated from log transformed values using the Unpaired t-test (two-tailed).
[d] MCW9632 Ura^- Ade^+ versus MCW9632 Ura^- Ade^-.
