# Supplementary material for: Gene duplication and deletion caused by over-replication at a fork barrier
Source: Nat Commun. 2023 Nov 25;14:7730. doi: 10.1038/s41467-023-43494-7 (PMC10676400; doi:10.1038/s41467-023-43494-7)
Supplement: Supplementary file 3 — Description of Additional Supplementary Files [file 41467_2023_43494_MOESM3_ESM.pdf]

Description of Additional Supplementary Files

File Name: Supplementary Data 1
Description: Table of recombination data, including recombination frequencies, strain numbers, relevant genotypes, the number of colonies tested for each strain (*n*), and relevant p-values. The data relate to Figures 1c, 2b, 2c, 2d, 3b, 3c, 3d, 4, 5 and 6, and Supplementary Figures 4, 6, 7 and 8.

File Name: Supplementary Data 2
Description: Details of the statistical analysis of the data in Figures 1 – 7, Supplementary Figures 4, 6, 7 and 8, Supplementary Data 1, and Supplementary Table 2.

File Name: Supplementary Data 3
Description: List of *S. pombe* strains used in the study, including strain numbers, genotypes and source.
