# Supplementary material for: Gene duplication and deletion caused by over-replication at a fork barrier
Source: Nat Commun. 2023 Nov 25;14:7730. doi: 10.1038/s41467-023-43494-7 (PMC10676400; doi:10.1038/s41467-023-43494-7)
Supplement: Supplementary file 5 — Reporting Summary [file 41467_2023_43494_MOESM5_ESM.pdf]

nature portfolio

Corresponding author(s): Matthew C. Whitby
Last updated by author(s): 2/11/2023

Reporting Summary

Nature Portfolio wishes to improve the reproducibility of the work that we publish. This form provides structure for consistency and transparency in reporting. For further information on Nature Portfolio policies, see our Editorial Policies and the Editorial Policy Checklist.

Statistics

For all statistical analyses, confirm that the following items are present in the figure legend, table legend, main text, or Methods section.

n/a	Confirmed	
☐	☒	The exact sample size (*n*) for each experimental group/condition, given as a discrete number and unit of measurement
☐	☒	A statement on whether measurements were taken from distinct samples or whether the same sample was measured repeatedly
☐	☒	The statistical test(s) used AND whether they are one- or two-sided<br>*Only common tests should be described solely by name; describe more complex techniques in the Methods section.*
☒	☐	A description of all covariates tested
☐	☒	A description of any assumptions or corrections, such as tests of normality and adjustment for multiple comparisons
☐	☒	A full description of the statistical parameters including central tendency (e.g. means) or other basic estimates (e.g. regression coefficient) AND variation (e.g. standard deviation) or associated estimates of uncertainty (e.g. confidence intervals)
☐	☒	For null hypothesis testing, the test statistic (e.g. *F*, *t*, *r*) with confidence intervals, effect sizes, degrees of freedom and *P* value noted<br>*Give P values as exact values whenever suitable.*
☒	☐	For Bayesian analysis, information on the choice of priors and Markov chain Monte Carlo settings
☒	☐	For hierarchical and complex designs, identification of the appropriate level for tests and full reporting of outcomes
☒	☐	Estimates of effect sizes (e.g. Cohen's *d*, Pearson's *r*), indicating how they were calculated

Our web collection on statistics for biologists contains articles on many of the points above.

Software and code

Policy information about availability of computer code

Data collection	Image Reader Version 2.02 (Fujifilm)
Data analysis	Image Gauge Version 4.21 (Fujifilm), Image Lab Version 6.1.0 build 7 (BioRad), Protos 3 software Version 1.2.4.0 (Synoptics Ltd), Excel for Mac Version 16.74 (Microsoft®) and GraphPad Prism Version 9.3.1 (GraphPad Software, San Diego, CA).

For manuscripts utilizing custom algorithms or software that are central to the research but not yet described in published literature, software must be made available to editors and reviewers. We strongly encourage code deposition in a community repository (e.g. GitHub). See the Nature Portfolio guidelines for submitting code & software for further information.

Data

Policy information about availability of data

All manuscripts must include a data availability statement. This statement should provide the following information, where applicable:

- Accession codes, unique identifiers, or web links for publicly available datasets
- A description of any restrictions on data availability
- For clinical datasets or third party data, please ensure that the statement adheres to our policy

All data generated or analysed during this study are included in this published article and its Supplementary Information file. The replication origins indicated in Figures 1a, 2a, 3a, 7a, and Supplementary Figures 3a and 5a are listed in OriDB (http://pombe.oridb.org). Source data are provided with this paper.

Research involving human participants, their data, or biological material

Policy information about studies with human participants or human data. See also policy information about sex, gender (identity/presentation), and sexual orientation and race, ethnicity and racism.

Reporting on sex and gender	Not applicable
Reporting on race, ethnicity, or other socially relevant groupings	Not applicable
Population characteristics	Not applicable
Recruitment	Not applicable
Ethics oversight	Not applicable

Note that full information on the approval of the study protocol must also be provided in the manuscript.

Field-specific reporting

Please select the one below that is the best fit for your research. If you are not sure, read the appropriate sections before making your selection.

☒ Life sciences ☐ Behavioural & social sciences ☐ Ecological, evolutionary & environmental sciences

For a reference copy of the document with all sections, see nature.com/documents/nr-reporting-summary-flat.pdf

Life sciences study design

All studies must disclose on these points even when the disclosure is negative.

Sample size	A minimum sample size for recombination experiments was calculated using the "A priori: Compute required sample size" power analysis tool in G*Power 3.1 (Faul, F., Erdfelder, E., Buchner, A., & Lang, A.-G., 2009, Behavior Research Methods, 41, 1149-1160), with an alpha value of 0.05, a power (1 - beta) value of 0.8 and an effect size (Cohen's d) of 1.2. Pilot recombination assays provided approximate mean values and standard deviations from which the effect size was calculated.
Data exclusions	No data were excluded from the analysis
Replication	During the construction of each yeast strain, at least three independent isolates with the same genotype were tested in pilot recombination assays. In all cases isolates with the same genotype exhibited the same recombination phenotype.
Randomization	All strains assayed for recombination were treated the same.
Blinding	Blinding is not relevant to this study as any potential bias in counting colonies in recombination assays was removed through the use of an automated colony counter.

Reporting for specific materials, systems and methods

We require information from authors about some types of materials, experimental systems and methods used in many studies. Here, indicate whether each material, system or method listed is relevant to your study. If you are not sure if a list item applies to your research, read the appropriate section before selecting a response.

Materials & experimental systems

n/a	Involved in the study	
☒	☐	Antibodies
☐	☒	Eukaryotic cell lines
☒	☐	Palaeontology and archaeology
☒	☐	Animals and other organisms
☒	☐	Clinical data
☒	☐	Dual use research of concern
☒	☐	Plants

Methods

n/a	Involved in the study	
☒	☐	ChIP-seq
☒	☐	Flow cytometry
☒	☐	MRI-based neuroimaging

Eukaryotic cell lines

Policy information about cell lines and Sex and Gender in Research

Cell line source(s)	The S. pombe strains used in this study are derivatives of the heterothallic strains 972 h- and 975 h+ isolated by Urs Leupold (Fantes & Hoffman, Genetics. 2016; 203: 621-629). They were obtained from existing laboratory stocks or generated by genetic crosses and/or targeted DNA integrations as detailed in the manuscript and associated supplementary material.
Authentication	The genotype of each S. pombe strain was verified by determining the presence of genetic markers and/or diagnostic PCR and DNA sequencing.
Mycoplasma contamination	Not applicable
Commonly misidentified lines (See ICLAC register)	Not applicable
